# Dynamic mitochondrial responses to a high-fat diet in ***Drosophila melanogaster***

**DOI:** 10.1038/s41598-018-36060-5

**Published:** 2019-03-14

**Authors:** Robert P. J. Cormier, Camille M. Champigny, Chloé J. Simard, Patrick-Denis St-Coeur, Nicolas Pichaud

**Affiliations:** Department of Chemistry and Biochemistry, Université de Moncton, Moncton, NB E1A 3E9 Canada

## Abstract

Mitochondria can utilize different fuels according to physiological and nutritional conditions to promote cellular homeostasis. However, during nutrient overload metabolic inflexibility can occur, resulting in mitochondrial dysfunctions. High-fat diets (HFDs) are usually used to mimic this metabolic inflexibility in different animal models. However, how mitochondria respond to the duration of a HFD exposure is still under debate. In this study, we investigated the dynamic of the mitochondrial and physiological functions in *Drosophila melanogaster* at several time points following an exposure to a HFD. Our results showed that after two days on the HFD, mitochondrial respiration as well as ATP content of thorax muscles are increased, likely due to the utilization of carbohydrates. However, after four days on the HFD, impairment of mitochondrial respiration at the level of complex I, as well as decreased ATP content were observed. This was associated with an increased contribution of complex II and, most notably of the mitochondrial glycerol-3-phosphate dehydrogenase (mG3PDH) to mitochondrial respiration. We suggest that this increased mG3PDH capacity reflects the occurrence of metabolic inflexibility, leading to a loss of homeostasis and alteration of the cellular redox status, which results in senescence characterized by decreased climbing ability and premature death.

## Introduction

A change in dietary resources is considered to be amongst the most important environmental stressors for an organism, impacting several aspects of phenotype. Both nutrient scarcity and abundance have most likely participated in shaping the evolution of cellular processes, and adjustments at the molecular and metabolic levels allowing to restore cellular homeostasis are crucial to survive these types of stress^1^. At the subcellular level, mitochondria integrate multiple metabolic pathways and produce the majority of adenosine triphosphate (ATP) by oxidative phosphorylation (OXPHOS), sustaining life itself. The mitochondrial machinery can modulate the utilization of different fuels such as glucose and fat, which enables a metabolic flexibility that is controlled by nutrient availability and physiological conditions^2^. However, during nutrient overload, a metabolic inflexibility characterised by competition between fuels such as carbohydrates and fatty acids can occur, resulting in a lessened ability to select proper mitochondrial substrates^2^. As a consequence, the cell fails to adjust fuel choice in response to nutritional demand resulting in impaired homeostasis and mitochondrial dysfunctions^2^. These mitochondrial dysfunctions are identified by a deficiency to oxidize substrates and/or to produce ATP which induce impaired fuel alternation and energy dysregulation^2^.

**Figure 1:**
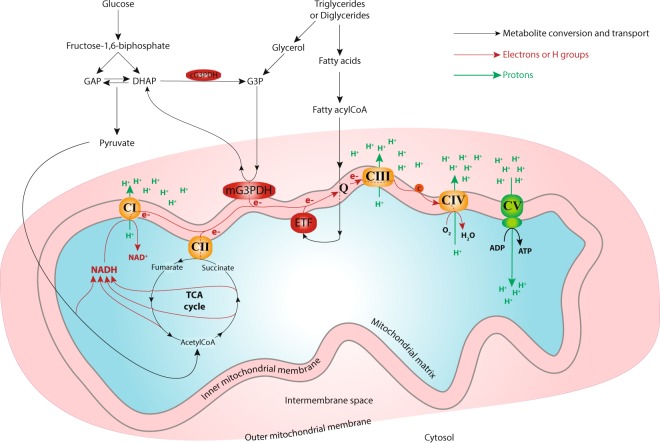
Schematic representation of mitochondrial metabolism. Carbohydrates such as glucose and fatty acids obtained from degradation of triglycerides and diglycerides are transformed in the cytosol before entering the mitochondria. Inside the mitochondrial matrix these substrates produce reducing equivalents (such as NADH or FADH_2_) that are used to transport the electrons from the mitochondrial complexes embedded in the mitochondrial inner membrane to the molecular oxygen. This transport allows the generation of a proton gradient which in turn powers the ATP synthase, generating ATP. AcetylCoA: acetylcoenzyme A; c: cytochrome c; CI: complex I; CII: complex II; CIII: complex III; CIV: complex IV; CV: complex V-ATPsynthase; cG3PDH: cytosolic glycerol-3-phosphate dehydrogenase; mG3PDH: mitochondrial glycerol-3-phosphate dehydrogenase; DHAP: dihydroxyacetone phosphate; e^−^: electrons; ETF: electron transport flavoprotein; G3P: glycerol-3-phosphate; GAP: glyceraldehyde-3-phosphate; H^+^: protons; Q: ubiquinone pool; TCA: tricarboxylic acid cycle.

Nutrients from the diet are absorbed, converted to substrates in the cell’s cytosol, and are then transported into the mitochondrial matrix^3–5^. Inside the mitochondrial matrix, substrate-derived metabolites are oxidized, leading to the formation of reducing equivalents such as nicotinamide adenine dinucleotide (NADH) and flavin adenine dinucleotide (FADH_2_), which are used to supply the electron transport system (ETS) located in the inner mitochondrial membrane^5,6^ (Fig. 1). Complex I, the main entry point of electrons into the ETS, oxidizes the mitochondrial NADH generated by the tricarboxylic acid cycle (TCA). Other complexes such as complex II and the electron transferring flavoprotein (ETF) oxidize the FADH_2_ produced by the TCA via succinate, and by fatty acid oxidation via fatty acylCoA, respectively^5,6^ (Fig. 1). Additionally, the mitochondrial glycerol-3-phosphate dehydrogenase (mG3PDH) allows the entry of electrons into the ETS through the reduction of glycerol-3-phosphate derived from either dihydroxyacetone phosphate (a metabolite from glycolysis) or from the glycerol obtained by triglyceride or diglyceride degradation^7,8^ (Fig. 1). The electrons from these different complexes are then sequentially transferred from complex III, to complex IV, before reaching the final acceptor, molecular oxygen. This electron transport generates a proton-motive force used by complex V to drive the phosphorylation of ADP to ATP (Fig. 1).

These different electron feeders (among others, see^9^) enable mitochondria to switch between oxidative substrates depending on their availability. A complex molecular network of effectors and transcriptional factors known as the nutrient sensing pathways is tightly linked to mitochondrial functions and allows the integration and the coordination of the organism’s metabolism through hormonal signals^1,10^. While these nutrient sensing pathways are under thorough investigation and are steadily being characterized in different physiological contexts, the modulation of mitochondrial responses according to nutrient availability is less understood. Nutrient overload has been linked to metabolic inflexibility in which mitochondrial dysfunctions play a central part, leading to a loss of homeostasis and the occurrence of several pathologies (reviewed in^2^). For example, chronic exposure to a high fat diet (HFD) has been shown to result in the modification of mitochondrial quantity and oxidative functions associated with obesity and insulin resistance in different model organisms^11–16^. Several studies have shown that in rodent skeletal muscle, mitochondrial functions are increased, decreased or unaffected depending on the exposure to different types of HFD^12,15–18^. These divergent results suggest that HFD composition as well as the duration of the exposure differently affect mitochondrial functions that adjust accordingly to promote metabolic homeostasis. However, animal models such as rats and/or mice can be problematic to study precise mitochondrial responses, as the experimental time-frame to evaluate the short- and long-term effects of a HFD can be difficult to determine.

Recently, the fruit fly, *Drosophila melanogaster*, has emerged as a suitable model to understand the fundamental mechanisms that control metabolism^19–24^. Drosophila fed a HFD display increased triglyceride fat, insulin resistance, deregulation of insulin-TOR signalling, oxidative stress, cardiac dysfunctions as well as alterations in fatty acid, amino acid and carbohydrate metabolisms^19,25–27^. However, to the best of our knowledge, no studies have investigated mitochondrial oxygen consumption changes in Drosophila fed on such diet at different experimental time points (but see^27^). In this study, we used this model to investigate the physiological and mitochondrial responses at different days following an exposure to a HFD. Specifically, we fed Drosophila either a standard diet (SD) or a HFD (SD supplemented with 20% (w/v) coconut oil as an increased source of saturated fat) and evaluated longevity, as well as climbing abilities, mitochondrial respiration, ATP content, glucose and glycogen concentrations, and enzymatic activities of pyruvate kinase (PK) and citrate synthase (CS) before (Day 0) and at several time points following the exposure to the HFD (Days 1, 2, 4 and 10). We hypothesized that after a short-term exposure to the HFD, adjustments of mitochondrial functions will occur to maintain cellular homeostasis. However, following long-term exposure, we suspected that the emergence of mitochondrial dysfunctions will result in loss of homeostasis and physiological dysfunctions, ultimately leading to premature death.

## Results

### Longevity and climbing abilities

**Figure 2:**
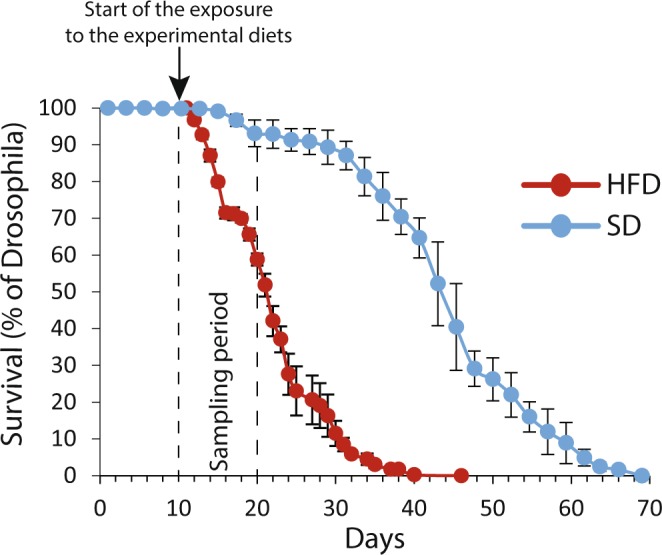
Survival curve of *Drosophila melanogaster* males exposed to either a standard diet (SD, blue) or a high-fat diet (HFD, red) at 10-day-old. Results are presented as the percentage of Drosophila alive counted every 1–2 days (for the HFD, N > 125) or every 2–3 days (for the SD, N = 150) after being raised for 10 days on the SD.

After hatching, flies were placed 10 days on the SD. They were then transferred to vials containing either the HFD or the SD, and the longevity was evaluated for each diet (Fig. 2). The HFD caused a significant reduction in lifespan (log-rank χ^2^ = 313, P < 0.001). Specifically, the median lifespan of flies fed with the HFD was decreased by 51.4% (total median lifespan of 45.3 and 22.0 days for flies on SD and HFD, respectively), and maximum lifespan was decreased by 33.3% (total maximum lifespan of 69.0 and 46.0 days for flies on SD and HFD, respectively). Interestingly, only 13% of flies were dead after 4 days of exposure to the HFD, while more than 40% were dead after 10 days of exposure.

**Figure 3:**
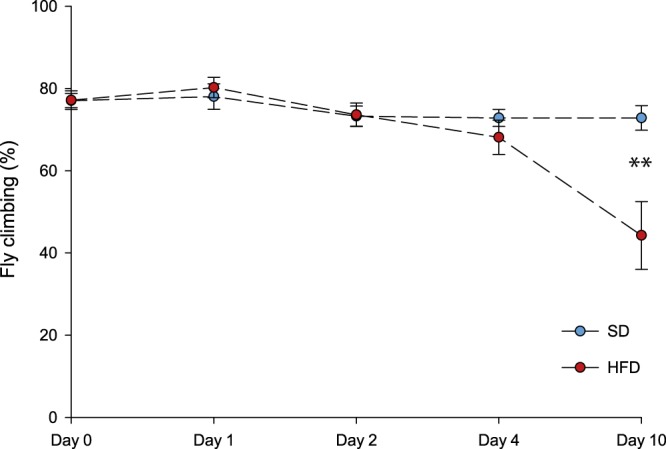
Climbing abilities of *Drosophila melanogaster* males exposed to either a standard diet (SD, blue) or a high-fat diet (HFD, red). The number of flies climbing in the top quarter of the vial was counted after 30 s for each day following the exposure to either the SD (blue, N > 40, in triplicates) or the HFD (N > 40 for Days 0–4 and N > 25 for Day 10, in triplicates). Stars depict significant differences between the SD and the HFD on each day of exposure to the diets (Student’s t-test): *P < 0.05, **P < 0.01, ***P < 0.001.

Climbing abilities were evaluated on flies fed either the SD or the HFD at days 0, 1, 2, 4 and 10 by counting the number of flies able to climb in the top quarter of a 9.4 cm tall × 2.5 cm wide empty vial after 30 s. Although flies exposed to the SD did not display any differences in climbing abilities for the duration of the experiment, the HFD caused a decrease in climbing abilities. Specifically, the climbing ability was significantly decreased at Day 10 in flies fed the HFD compared to the ones fed the SD (Student’s t-test, P = 0.004; Fig. 3). This indicates that the HFD caused an impairment of physiological functions measurable after 10 days of the exposure.

### Respiration rates, mitochondrial ratios and ATP content in thorax

**Figure 4:**
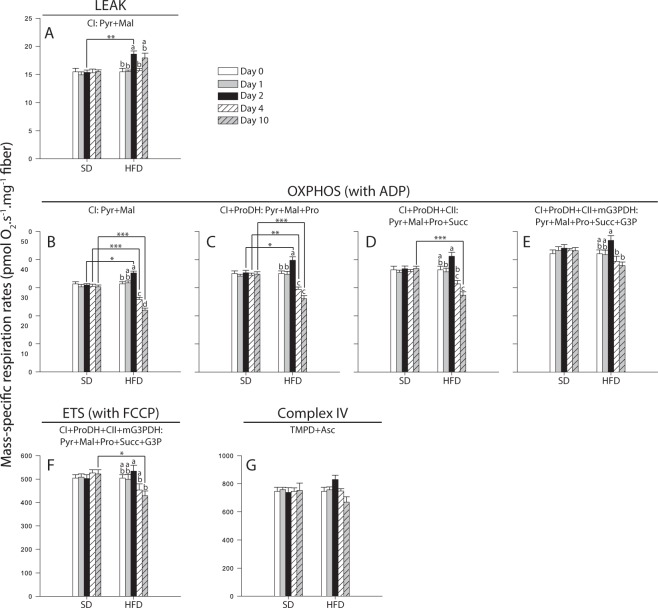
Mass-specific respiration rates measured in permeabilized thorax of *Drosophila melanogaster* males exposed to either a standard diet (SD) or a high-fat diet (HFD). Mitochondrial oxygen consumption was measured in presence of (**A**) pyruvate + malate (CI-LEAK), (**B**) + ADP (CI-OXPHOS), (**C**) + proline (CI + ProDH-OXPHOS), (**D**) + succinate (CI + ProDH + CII-OXPHOS), (**E**) + glycerol-3-phosphate (CI + ProDH + CII + mG3PDH-OXPHOS), (**F**) + FCCP (CI + ProDH + CII + mG3PDH-ETS), as well as after inhibition of complexes I, II and III by rotenone, malonate and antimycin A, respectively, and addition of (**G**) TMPD + ascorbate (Complex IV). Results are means ± s.e.m for each day of the exposure and for each diet (N = 6), and have been analyzed using a two-way ANOVA followed by pairwise comparisons of the least-squares means using adjusted P-values (Tukey method). Stars depict significant differences between the SD and the HFD on each day of exposure with: *P < 0.05, **P < 0.01, ***P < 0.001. Dissimilar lowercase letters denote changes between days of exposure to a specific diet, with each different letter(s) representing statistically significant differences (P < 0.05). Asc: ascorbate; CI: complex I; CII: complex II; CIV: complex IV;; FCCP: carbonyl cyanide 4-(trifluoromethoxy)phenylhydrazone; G3P: glycerol-3-phosphate; mG3PDH: mitochondrial glycerol-3-phosphate dehydrogenase; Pyr + Mal: pyruvate + malate; Pro: proline; ProDH: proline dehydrogenase; Succ: succinate; TMPD: N,N,N′,N,-Tetramethyl-p-phenylenediamine.

Measurement of mitochondrial oxygen consumption at 24 °C (N = 6 for each day) were performed on permeabilized thorax using a high-resolution respirometer Oxygraph-O2K (Oroboros Instruments, Innsbruck, Austria), as previously described^28–30^. Respiration rates were obtained after injection of different substrates: pyruvate + malate (CI-LEAK; Fig. 4A), +ADP (CI-OXPHOS; Fig. 4B), +proline (CI + ProDH-OXPHOS; Fig. 4C), +succinate (CI + ProDH + CII-OXPHOS; Fig. 4D), +glycerol-3-phosphate (CI + ProDH + CII + mG3PDH-OXPHOS; Fig. 4E), +FCCP (CI + ProDH + CII + mG3PDH-ETS; Fig. 4F), +rotenone + malonate + antimycin A (residual oxygen consumption; Fig. S1), and +TMPD + ascorbate (Complex IV; Fig. 4G).

CI-LEAK, CI-OXPHOS, CI+ ProDH-OXPHOS, and CI + ProDH + CII-OXPHOS respiration rates were affected by the Day, the Diet and the interaction Day × Diet (two-way ANOVA, Table 1). CI-LEAK corresponds to the mitochondrial oxygen consumption, which compensates for the proton leak through the inner mitochondrial membrane without ADP phosphorylation. Under the HFD, a significant increase was observed at Day 2 compared to Days 0, 1 and 4 (P-values ≤ 0.017, respectively; Fig. 4A). For Day 2, CI-LEAK was also higher for flies fed the HFD compared to those fed the SD (P = 0.005; Fig. 4A). When ADP was added to trigger phosphorylation of ATP (CI-OXPHOS), a significant increase was observed in flies fed the HFD between Day 0 and Day 2 (P = 0.039; Fig. 4B). Under the same diet, CI-OXPHOS was significantly decreased at Day 4 compared to Days 0, 1 and 2 (all P-values < 0.001), and was further decreased at Day 10 (P-values ≤ 0.0012 compared to Day 4; Fig. 4B). Moreover, flies fed the HFD had higher CI-OXPHOS at Day 2 (P = 0.017), but lower rates at Day 4 and Day 10 compared to the SD (P-values < 0.001). For the HFD, a significant increase was observed when proline was added (CI + ProDH-OXPHOS) at Day 2 (P-values ≤ 0.020), followed by a significant decline at both Day 4 (P-values ≤ 0.006) and Day 10 (all P-values < 0.001; Fig. 4C). Again, when comparing diets, flies on the HFD had higher CI + ProDH-OXPHOS rates at Day 2 (P = 0.039), but lower rates at Days 4 and 10 (P ≤ 0.010; Fig. 4C). For the HFD, the same pattern was observed using succinate, with CI + ProDH + CII-OXPHOS being increased on Day 2 when compared to Day 1 (P = 0.029 when compared to Day 1), but decreased at Day 4 (P < 0.001 when compared to Day 2), and at Day 10 (when compared to Days 0, 1 and 2, all P-values < 0.001; Fig. 4D). However, flies exposed to the HFD had higher CI + ProDH + CII-OXPHOS only at Day 10 when compared to the SD. Altogether, this suggests that mitochondrial oxygen consumption is affected at the level of complex I, with increased respiration rates after two days of exposure, but decreased respiration rates from four days of exposure to the HFD.

**Table 1:** Analyses of variance showing F-values for mitochondrial respiration rates, mitochondrial ratios and ATP content measured in thorax of *Drosophila melanogaster* males exposed to either a standard diet or a high-fat diet.

	Denominator degrees of freedom	Day (degrees of freedom = 4)	Diet (degrees of freedom = 1)	Day × Diet (degrees of freedom = 4)
**Respiration rates**				
CI-LEAK	50	4.22**	14.70***	3.30*
CI-OXPHOS	50	31.99***	16.06***	26.80***
CI + ProDH-OXPHOS	50	17.84***	8.98**	15.68***
CI + ProDH + CII-OXPHOS	50	11.31***	6.70*	11.23***
CI + ProDH + CII + mG3PDH-OXPHOS	50	3.68*	3.37	2.71*
CI + ProDH + CII + mG3PDH-ETS	50	1.48	5.95*	3.88**
Complex IV	50	1.45	0.01	1.96
**Mitochondrial ratios**				
P/L for complex I	50	21.15***	43.37***	14.37***
Contribution of proline	50	1.96	0.0006	0.69
Contribution of succinate	50	14.36***	8.27**	8.10***
Contribution of G3P	50	14.70***	6.51*	18.55***
ATP content	20	34.89***	0.28	38.53***

*P < 0.05, **P < 0.01, ***P < 0.001.

CI + ProDH + CII + mG3PDH-OXPHOS was influenced by the Day and the interaction Day × Diet (two-way ANOVA, Table 1). Specifically, a slight but non-significant increase was observed on Day 2 (Fig. 4E). For Days 4 and 10, significantly decreased CI + ProDH + CII + mG3PDH-OXPHOS were detected, but only when compared to Day 2 (P-values ≤ 0.012; Fig. 4E). However, no significant differences were detected when comparing the SD and the HFD for each day of the exposure (Fig. 4E). This suggests that increased capacity of the mG3PDH allowed to partially compensate for the defect observed with the previous respiration rates. The non-coupled respiration (CI + ProDH + CII + mG3PDH-ETS) was affected by the Diet and the interaction Day × Diet (two-way ANOVA, Table 1). This respiration rate were similar across the days of exposure for flies fed the HFD, except for a decrease observed at Day 10 when compared to Day 2 (P = 0.088; Fig. 4F). A significant decrease was also detected for this rate at Day 10 when comparing the SD and the HFD (P = 0.029; Fig. 4F). Finally, complex IV was not influenced by any of the fixed factors nor their interaction (two-way ANOVA, Table 1; Fig. 4G).

**Figure 5:**
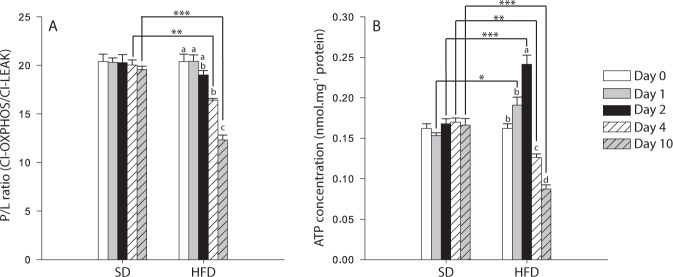
Mitochondrial coupling and ATP content measured in thorax muscle of *Drosophila melanogaster* males exposed to either a standard diet (SD) or a high-fat diet (HFD). (**A**) P/L ratio at the level of complex I (P/L = CI-OXPHOS/CI-LEAK), as an indicator of mitochondrial quality and of mitochondrial coupling (N = 6), (**B**) ATP content (N = 3). Results are means ± s.e.m for each day of the exposure and for each diet, and have been analyzed using a two-way ANOVA followed by pairwise comparisons of the least-squares means using adjusted P-values (Tukey method). Stars depict significant differences between the SD and the HFD on each day of exposure with: *P < 0.05, **P < 0.01, ***P < 0.001. Dissimilar lowercase letters denote changes between days of exposure to a specific diet, with each different letter(s) representing statistically significant differences (P < 0.05).

When flies were exposed to the HFD, the P/L ratio (calculated as CI-OXPHOS/CI-LEAK; Fig. 5A) which is an indicator of the coupling efficiency at the level of complex I, declined at Day 4 (P < 0.001 when compared to Day 0 and 1), and was further decreased at Day 10 (all P-values < 0.001 when compared to Days 0, 1, 2 and 4). We also detected significant differences between diets at Day 4 (P = 0.002) and Day 10 (P < 0.001), indicating possible mitochondrial dysfunctions. For ATP content measured in thorax muscle, the same pattern as most respiration rates was observed in flies exposed to the HFD (Fig. 5B). Specifically, a significant increase was detected at Day 2 (P-values ≤ 0.002 for comparisons with Day 0 and Day 1), followed by a significant decline at Day 4 (P-values ≤ 0.040 when compared to Days 0, 1, and 2, respectively) that was even more pronounced at Day 10 (P < 0.001 compared to Days 0–2, and P = 0.021 compared to Day 4; Fig. 5B). Moreover, significant differences were also detected between the SD and the HFD (P-values ≤ 0.029 for Days 1 to 10; Fig. 5B), confirming mitochondrial dysfunctions and energy dysregulation.

**Figure 6:**
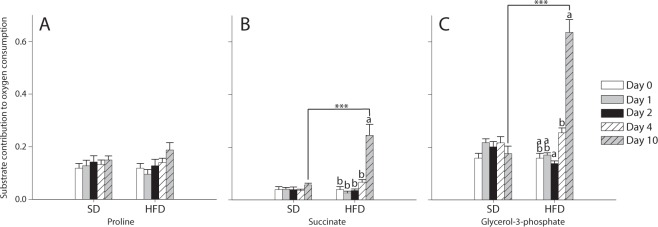
Contribution of different substrates to the mitochondrial oxygen consumption of *Drosophila melanogaster* males exposed to either a standard diet (SD) or a high-fat diet (HFD). (**A**) contribution of proline = (CI + ProDH-OXPHOS − CI-OXPHOS)/CI-OXPHOS; (**B**) contribution of succinate = (CI + ProDH + CII-OXPHOS − CI + ProDH-OXPHOS)/CI + ProDH-OXPHOS; (**C**) contribution of G3P = (CI + ProDH + CII + mG3PDH-OXPHOS − CI + ProDH + CII-OXPHOS)/CI + ProDH + CII-OXPHOS. Results are means ± s.e.m for each day of the exposure and for each diet (N = 6) and have been analyzed using a two-way ANOVA followed by pairwise comparisons of the least-squares means using adjusted P-values (Tukey method). Stars depict significant differences between the SD and the HFD on each day of exposure with: *P < 0.05, **P < 0.01, ***P < 0.001. Dissimilar lowercase letters denote changes between days of exposure to a specific diet, with each different letter(s) representing statistically significant differences (P < 0.05).

At each day of interest, we also calculated the contribution of: proline = (CI + ProDH-OXPHOS − CI-OXPHOS)/CI-OXPHOS; succinate = (CI + ProDH + CII-OXPHOS − CI + ProDH-OXPHOS)/CI + ProDH-OXPHOS; and G3P = (CI + ProDH + CII + mG3PDH-OXPHOS − CI + ProDH + CII-OXPHOS)/CI + ProDH + CII-OXPHOS. No effects of the Day, the Diet nor of the interaction Day × Diet were detected on the contribution of proline to the oxygen consumption (Fig. 6A), but the contributions of succinate and G3P were significantly affected by all these factors (two-way ANOVA, Table 1; Fig. 6B,C). Under the HFD, contribution of succinate was significantly augmented at Day 10 (all P-values < 0.001 when compared to Days 0–4; Fig. 6B). Contribution of G3P was significantly increased in flies exposed to the HFD at Day 4 (P = 0.009 when compared to Day 2), and was further increased at Day 10 (all P-values < 0.001 with all the other days; Fig. 6C). This significant increase was also observed at Day 10 when comparing the SD with the HFD (P < 0.001), confirming the possible compensation by the mG3PDH.

### Glycogen and glucose contents in whole fly homogenates

**Figure 7:**
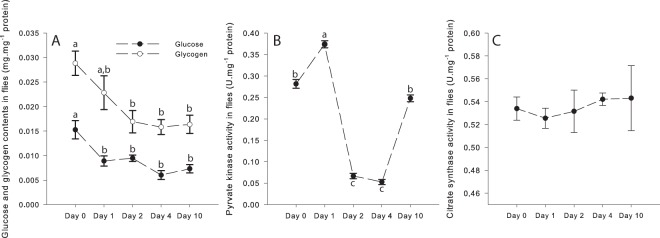
Effects of the high-fat diet (HFD) on (**A**) Glucose and Glycogen contents of flies, (**B**) Pyruvate kinase activity, and (**C**) Citrate synthase activity. Results are means ± s.e.m for each day of the exposure (N = 4–5 for glucose and glycogen contents; N = 6 for pyruvate kinase and citrate synthase activities) and have been analyzed using a one-way ANOVA followed by pairwise comparisons of the least-squares means using adjusted P-values (Tukey method). Dissimilar lowercase letters denote changes between days of exposure to a specific diet, with each different letter(s) representing statistically significant differences (P < 0.05).

The HFD exposure had effects on both glucose and glycogen contents (one-way ANOVA; Fig. 7A). For glucose, significant decreases were detected for Days 1, 2, 4 and 10 compared to Day 0 (P-values ≤ 0.015). For glycogen, the same trend was observed, with a slight decrease from Day 1 but only significant between Day 0 and Days 2, 4 and 10 (P-values ≤ 0.018). This suggests depletion of the main sources of carbohydrates following the HFD exposure.

### Enzymatic activities in whole fly homogenates

PK and CS activities were measured in whole fly homogenates and were analysed with a one-way ANOVA. PK was determined to evaluate the pyruvate formation from the glycolytic pathway and was significantly increased at Day 1 (P < 0.001 compared to Day 0), but decreased at Day 2 and Day 4 (P < 0.001 compared to either Day 0 or Day 1; Fig. 7B). At Day 10, PK activity was similar to Day 0 (Fig. 7B). CS activity was used as a proxy for mitochondrial density^31,32^ and was not influenced by the HFD exposure with values similar across the different days of exposure (Fig. 7C).

## Discussion

In this study, we evaluated the mitochondrial and physiological responses of *Drosophila melanogaster* following an exposure to a HFD characterised by an increased content in saturated fatty acids compared to a SD. Our results showed that at the mitochondrial level, increased respiration rates driven by an increased capacity of complex I were observed after two days on the HFD which is likely due to utilization of carbohydrates despite the abundance of fatty acids, leading to adjustments of mitochondrial metabolism to maintain metabolic homeostasis and higher ATP content in the thorax muscle. However, after four days on the HFD, an impairment of the mitochondrial respiration at the level of complex I was observed which was associated to a decrease P/L ratio and in ATP content, as well as an increased contribution of complex II and mG3PDH to mitochondrial respiration. These increased contributions reflect the occurrence of metabolic inflexibility, especially after 10 days, leading to a loss of homeostasis which results in physiological defects and senescence.

Nutrient availability is known to influence metabolic homeostasis and transitions between different substrate utilization by the cell occurs during normal energy metabolism^2,8,33^. However, it has been suggested that substrate competition at the mitochondrial level during nutrient overload can lead to metabolic deregulation and mitochondrial impairment which may be at the origin of several pathological conditions^1,2,34–36^. As a matter of fact, HFDs with high caloric content have been widely used to induce metabolic inflexibility and to study metabolic diseases such as type 2 diabetes and obesity-related disorders in several mammals^11,12,14,15^. Although it is generally accepted that a long-term increased fat intake might lead to mitochondrial defects (reviewed in^14^), how the mitochondria dynamically respond to nutrient overload caused by an exposure to a HFD is still a question under debate. It has been shown that mitochondrial proteins, fat oxidation capacity, mitochondrial respiration, and expression of genes involved in OXPHOS in rodent skeletal muscle are differently modulated in response to different HFDs^12^. These discrepancies could likely be attributed to different factors such as the animal model (rat vs mice), the diet composition in macronutrients or the fat source (animal vs vegetal, saturated vs unsaturated fatty acids), as well as the duration of the exposure.

To evaluate the effects of a HFD in a time relevant manner, we used *Drosophila melanogaster*, an animal model with a relatively short lifespan that has been shown to display metabolic inflexibility after exposure to HFD^19,23,25–27^. When Drosophila are fed a HFD enriched with 20% coconut oil, it has been shown that triglyceride levels as well as total fatty acid abundance were increased in Drosophila after seven days which was associated with a drastically reduced lifespan likely due to impaired metabolism^26,27^. Consistent with these studies, our results showed that the same HFD also induced an important decrease in lifespan. As most catabolic processes (carbohydrates, amino acids, and fatty acids) converge into the mitochondria for the production of ATP, we investigated mitochondrial oxygen consumption as well as ATP content in the thorax muscle. Our results showed that after two days on the HFD, mitochondrial oxygen consumption was increased using pyruvate, the end product of glycolysis, in combination with malate, an intermediate of the TCA cycle (CI-OXPHOS). The utilization of these substrates reflects an increased capacity of complex I to oxidize the NADH produced from pyruvate oxidation and the TCA cycle, which leads to increased ATP content. In Drosophila, carbohydrates are the main macronutrient sustaining mitochondrial metabolism in muscles^37^. Therefore, our results suggest that Drosophila are increasing their capacity to oxidize carbohydrates despite the abundance of fatty acids during the first days of the HFD exposure. This is confirmed with increased PK enzymatic activity at Day 1, as well as with decreased glucose content from Day 1, and decreased glycogen content from Day 2. Consistent with this hypothesis, glucose levels of flies exposed to either 20% or 30% coconut oil HFD for seven and two days, respectively, are significantly decreased suggesting utilization and depletion of carbohydrates during the first days of the exposure^19,26^. In parallel, the mitochondrial coupling (P/L ratio) is not affected and remains constant for the first two days of the HFD exposure, suggesting that mitochondrial homeostasis is maintained and that the increase observed represents efficient adjustments to cope for the dietary change. However, the capacity of the ETS to utilize the other substrates (proline, succinate and G3P) is unaffected.

After this initial adjustment period, CI-OXPHOS is significantly decreased at Day 4 and even more reduced at Day 10, which resulted in a concomitant decrease in the P/L ratio. Although this decrease could be attributed to decreased mitochondrial number^14–16^, we did not detect any changes of CS enzymatic activity or of Complex IV capacity, which are considered good markers of mitochondrial density^31,32^. This is in accordance with Trindade de Paula *et al*.^27^, who showed that Drosophila on a 20% HFD displayed decreased activity of dehydrogenases as well as mitochondrial viability after seven days of exposure, which was also associated with decreased climbing abilities and reduced lifespan. A possible explanation for these results is that fatty acids are efficiently stored on the short-term, but are then transported to the muscle cells and transformed into acetylcoenzyme A (Ac-CoA) for ATP generation, leading to inhibition of the pyruvate dehydrogenase complex (PDH), converting pyruvate to Ac-CoA. This is in accordance with the PK decreased enzymatic activity observed from Day 2, whereas it was increased at Day 1. Moreover, we detected increased capacity of complex II to utilize succinate which is formed by the TCA from Ac-CoA at Day 10. This increase was however not observed at Day 4, when CI-OXPHOS was starting to decrease. Interestingly, mG3PDH capacity to oxidize G3P was also importantly increased at both Days 4 and 10. G3P is a substrate that can be formed by two different pathways. First, it can be produced by glycolysis via conversion of dihydroxyacetone phosphate by the cytosolic G3PDH^7,38^. Inhibition of PDH by fatty acids should result in pyruvate accumulation, which was demonstrated after 7 days on a 20% coconut oil HFD^26^. In turn, this accumulation should promote increased metabolite concentrations upstream of glycolysis, including dihydroxyacetone phosphate, and thus, increased G3P should be available for mitochondrial respiration. G3P can also be formed via phosphorylation of the glycerol derived from triglyceride or diglyceride catabolism by the glycerol kinase^7,38^. Therefore, exposure to the HFD should result in higher G3P production, explaining the important increased contribution of G3P to the mitochondrial respiration.

Thus, a metabolic reprogramming seems to occur at the mitochondrial level after four days on the HFD, mainly driven by decreased pyruvate formation (as seen with PK activity) and oxidation leading to a decreased complex I capacity, and an increased capacity to oxidize G3P by the mG3PDH. Interestingly, it has recently been shown that after seven days on a 20% coconut oil HFD, increased reactive oxygen species (ROS) levels and lipid peroxidation were observed in Drosophila, suggesting the occurrence of oxidative stress that was associated with decreased lifespan and climbing abilities^27^. The mG3PDH has been shown to be an important site of superoxide production in both mammals and Drosophila^8,39–43^. Moreover, when respiring with G3P as a substrate, mitochondria from Drosophila are producing higher rate of superoxide than with other substrates^42^. The increased mG3Pdh capacity observed in our study suggests that after four days, higher superoxide rates are produced, possibly leading to oxidative damages. In turn, these damages could explain the decreased lifespan observed, as higher reactive oxygen species production and oxidative damages are associated with aging^44^. This observed premature senescence occurs after the decreased oxygen consumption was detected, indicating that mitochondrial dysfunctions could be the cause of the reduced lifespan. We therefore suggest that the loss of homeostasis observed after four days on the HFD might be in part due to the mG3PDH capacity. Theoretically, an increased capacity of mG3PDH might help the cell to maintain homeostasis by providing an alternate route for both carbohydrates and fatty acids, diminishing metabolic inflexibility. However, this higher capacity might lead to increased reactive oxygen species production, altering the redox status of the cell and participating in the loss of homeostasis which leads to decreased ATP production, impairment of physiological functions, and premature death. This hypothesis remains to be confirmed, but characterization of mG3PDH regulation following a HFD exposure, as well as specific reactive oxygen species production by the mG3PDH using novel inhibitors of mG3PDH^41,45^ represents interesting research avenues for further studies.

In summary, our study reveals important dynamic changes of mitochondrial functions during the course of a HFD exposure in *Drosophila melanogaster*. On the short-term, mitochondrial functions and ATP content of the thorax muscle are increased, which likely represents an efficient adjustment to a change in dietary resources. However, after a few days, metabolic inflexibility occurs, likely due to accumulation of fatty acids and depletion of carbohydrates, leading to mitochondrial dysfunctions at the level of complex I, decreased ATP content and loss of homeostasis, which in turn cause physiological defects and decreased lifespan. This provides a potential explanation for the contradicting results obtained in other animal models exposed to a HFD, as the duration of the exposure and the timing of the experiments used with these models might not be able to identify these dynamic changes. We also showed that mG3PDH might be a key player in the mitochondrial response to a HFD. Drosophila are increasingly used as a relevant model to study the underlying mechanisms of several metabolic diseases such as type 2 diabetes and obesity-related disorders^20,21,24,46,47^. Therefore, the mechanistic link between increased mG3PDH capacity and loss of homeostasis following the HFD exposure could provide significant new insights for the understanding of these diseases.

## Methods

### Drosophila model, longevity experiments and climbing assay

*Drosophila melanogaster* w^11,18^ (Bloomington Drosophila Stock Center, Bloomington, IN, USA) were reared at constant temperature (24.0 ± 0.1 °C), humidity (50% relative humidity), and diurnal cycle (12:12 h light:dark) on a standard cornmeal medium (see Supplementary material). Males were collected on the day they hatch and were transferred to new vials with the SD for 10 days. Afterwards, they were transferred to vials containing either a HFD (SD supplemented with 20% w/v coconut oil) shown to induce metabolic inflexibility^19,26,27^ or to new vials containing the SD, at constant densities (15 flies per vial). The fatty acid composition of the coconut oil used is presented in Table S1 (see Supplementary material).

Longevity was evaluated by recording the number of flies alive every 1–2 days after the transfer to the HFD (N > 125) or every 2–3 days after transfer to the SD (N = 150) and the experiments were repeated three times. Flies were transferred to fresh food every 2–5 days. For the climbing assay, flies were sampled at different days before (Day 0) and during exposure to the HFD (Days 1, 2, 4 and 10). Flies from the control group (SD) were also collected at the same days. Climbing ability (negative geotaxis) was measured as previously described^48^. Briefly, 15 flies were transferred to empty vials (9.4 cm tall × 2.5 cm wide) and were tapped to the bottom of the vial (N > 40 for Days 0–4 and N > 25 for Day 10 on the HFD; N > 40 for each day on the SD). The number of flies in the top quarter of the vial was counted after 30 s of climbing. These assays were repeated three times for each vial.

### Biochemical experiments

Following 0, 1, 2, 4, and 10 days of exposure to the HFD or to the SD, the heads and abdomens were dissected from the flies, and the resulting thorax were either directly processed for mitochondrial respiration or frozen in liquid nitrogen and stored at −80 °C for further measurements of ATP content. On sampling days whole flies were also collected, put on ice, rinsed two times in ice-cold phosphate-buffered saline (PBS), and frozen in liquid nitrogen for measurements of glucose and glycogen contents as well as for pyruvate kinase (PK) and citrate synthase (CS) enzymatic activities. ATP, glucose and glycogen contents as well as activities of pyruvate kinase (PK) and citrate synthase (CS) were normalized by total protein content which was measured using the bicinchoninic acid method^49^.

#### Mitochondrial oxygen consumption in permeabilized thorax

Permeabilization of thorax and measurement of mitochondrial oxygen consumption at 24 °C (N = 6 for each day) were performed as previously described^28–30^ (see Supplementary material). Permeabilized thorax were weighed and transferred into each chamber of an Oxygraph-O2K (Oroboros Instruments, Innsbruck, Austria) filled with air-saturated respiration medium with pyruvate (10 mM) and malate (2 mM). The oxygen concentration was raised to ~ 150% oxygen air-saturation (to avoid diffusion limitation in the tissues), the chambers were closed and the signal was allowed to stabilize in order to measure the LEAK respiration at the level of complex I (CI-LEAK). Injection of ADP (5 mM) enabled the measurement of mitochondrial oxygen consumption when the transport of electrons from complex I is coupled to the phosphorylation of ADP to ATP (CI-OXPHOS). These respiration rates were used to calculate the P/L ratio at the level of complex I (CI-OXPHOS/CI-LEAK), indicative of mitochondrial quality and of mitochondrial coupling^50^.

Functional integrity of the outer mitochondrial membrane was then verified by addition of cytochrome c (15 μM, results shown in supplementary material)^51^. Subsequent injection of several substrates were then performed: proline (5 mM), an important substrate for mitochondrial respiration in insects^52^ which provides electrons to the ETS via the proline dehydrogenase (CI + ProDH-OXPHOS); succinate (20 mM) which brings electrons to the ETS through complex II (CI + ProDH + CII-OXPHOS); and G3P (15 mM) that allows the transport of electrons to the ETS via the mG3PDH (CI + ProDH + CII + mG3PDH-OXPHOS). The respiration rates measured with these different substrates were used to evaluate the contribution of each substrate to mitochondrial oxygen consumption. At each day of interest, we calculated the contribution of: proline = (CI + ProDH-OXPHOS − CI-OXPHOS)/CI-OXPHOS; succinate = (CI + ProDH + CII-OXPHOS − CI + ProDH-OXPHOS)/CI + ProDH-OXPHOS; and G3P = (CI + ProDH + CII + mG3PDH-OXPHOS − CI + ProDH + CII-OXPHOS)/CI + ProDH + CII-OXPHOS.

Injection of an uncoupler, carbonyl cyanide 4-(trifluoromethoxy)phenylhydrazone (FCCP, steps of 0.5–1 μM), was then performed to measure the non-coupled respiration i.e. the non-phosphorylating respiration stimulated to maximal oxygen consumption (CI + ProDH + CII + mG3PDH-ETS). Subsequent inhibitions of complexes I, II and III by rotenone (0.5 μM), malonate (5 mM) and antimycin A (2.5 μM) were performed to evaluate the residual oxygen consumption which was used to correct the previous respiration rates measured. No differences in residual oxygen consumption were detected between the diets or between the different days of exposure (Fig. S1). Finally, ascorbate (2 mM) and N,N,N′,N,-Tetramethyl-p-phenylenediamine (TMPD, 0.5 mM) were added to evaluate the maximum capacity of complex IV (CIV), which was corrected for auto-oxidation of TMPD after inhibition of complex IV by sodium azide (20 mM). All measurements are presented as means of mass-specific respiration rates expressed as pmol O_2_.s^−1^.mg^−1^ of permeabilized fibers ± s.e.m.

#### ATP content in thorax muscle

Seven thorax were homogenized in 5 mM Tris, 137 mM NaCl, 2.7 mM KCl, 0.1% v/v triton X-100, pH 7.0. The ATP content was measured using an ATP Determination Kit (Molecular Probes^TM^, Eugene, OR, USA) according to the manufacturer’s protocol (N = 3). Briefly, ATP was quantified on triplicates using recombinant firefly luciferase and its substrate D-luciferin, and the resulting light was measured with a Varioskan^TM^ microplate reader (ThermoScientific^TM^) by luminometry. ATP content of the thorax is expressed as means of pmol of ATP per mg of proteins ± s.e.m.

#### Glycogen and glucose contents in whole flies

Seven flies were homogenized in ice-cold PBS and glucose and glycogen contents (N = 4–5) were measured using a Glucose Oxidase Assay kit (Sigma-Aldrich®), according to the manufacturer’s protocol at 540 nm. Glycogen was determined by measuring glycogen + glucose content in presence of amyloglucosidase and subtracting the glucose concentration (samples without amyloglucosidase) as described elsewhere^46^. Glucose and glycogen contents are expressed as means of μg of glucose or glycogen per mg of proteins ± s.e.m.

#### Enzymatic activities in whole flies

Seven flies were homogenized in 50 mM imidazole-HCl buffer, pH 7.8. PK activity was determined following the disappearance of NADH at 340 nm (ε = 6.22 mL.cm^−1^.μmol^−1^) for 4 min in a reaction medium containing 50 mM imidazole-HCl, 10 mM MgCl2, 100 mM KCl, 5 mM ADP, 0.15 mM NADH, 5 mM phosphoenolpyruvate and 0.6 U.mL^−1^ lactate dehydrogenase. CS activity was measured at 412 nm for 4 min following the reduction of 5,5′-dithiobis-2-nitrobenzoic acid (DTNB, ε = 13.6 mL.cm^−1^.μmol^−1^) using a 50 mM imidazole-HCl buffer containing 0.1 mM DTNB, 0.1 mM acetyl-CoA and 0.15 mM oxaloacetic acid. Both enzymatic activities are expressed as U.mg^−1^ protein, where U represents 1 μmol of substrate transformed to product in 1 min.

### Statistical analysis

All statistical analyses were performed with R software (version 3.1.0, Free Software Foundation, Boston, MA, USA). For longevity, a log-rank test was performed to detect survival differences between the SD and the HFD. For climbing assays, a Student’s t-test was performed to determine specific differences between the SD and the HFD at each day of exposure. For mitochondrial respiration rates, mitochondrial ratios, and ATP content, the data were fitted to a linear model and were analyzed using a two-way ANOVA with the day of exposure (Day) and the experimental diet (Diet) as fixed factors. If an interaction effect (Day*Diet) was detected, multiple comparisons were then tested with pairwise comparisons of the least-squares means using adjusted P-values (Tukey method). For glycogen and glucose contents, as well as for enzymatic activities, the data were fitted to a linear model and were analyzed using a one-way ANOVA with the day of exposure as fixed factor, followed with pairwise comparisons of the least-squares means. Significance was set at P < 0.05. For the ANOVAs, normality was verified with the Shapiro-Wilk’s test and homogeneity of variances was verified using the Levene’s test, and data were transformed when required.

## Electronic supplementary material

Supplementary information

Dataset 1

## Data Availability

The datasets for this manuscript have been uploaded as supplementary material.
