# Author Correction: Dynamic mitochondrial responses to a high-fat diet in *Drosophila melanogaster*

**DOI:** 10.1038/s41598-021-91650-0

**Published:** 2021-06-11

**Authors:** Robert P. J. Cormier, Camille M. Champigny, Chloé J. Simard, Patrick-Denis St-Coeur, Nicolas Pichaud

**Affiliations:** Department of Chemistry and Biochemistry, Université de Moncton, Moncton, NB E1A 3E9 Canada

Correction to: *Scientific Reports* 10.1038/s41598-018-36060-5, published online 14 March 2019

The original version of this Article contained an error in the Results and Methods section. The experiments measuring glycogen and glucose content, as well as enzymatic activities, were reported to have been performed on whole fly homogenates, however, these experiments were performed on thorax muscle homogenates.

Therefore, in the Results section, the subheading,

“Glycogen and glucose contents in whole fly homogenates”

now reads,

“Glycogen and glucose contents in thorax muscle homogenates”

The subheading,

“Enzymatic activities in whole fly homogenates”.

now reads,

“Enzymatic activities in thorax muscle homogenates”.

And the text,

“PK and CS activities were measured in whole fly homogenates and were analysed with a one-way ANOVA.”

now reads,

“PK and CS activities were measured in thorax muscle homogenates and were analysed with a one-way ANOVA.”

In the Methods, the subheading,

“Glycogen and glucose contents in whole flies”

now reads,

“Glycogen and glucose contents in thorax muscle”

The subheading,

“Enzymatic activities in whole flies”.

now reads,

“Enzymatic activities in thorax muscle”.

The text,

“Following 0, 1, 2, 4, and 10 days of exposure to the HFD or to the SD, the heads and abdomens were dissected from the flies, and the resulting thorax were either directly processed for mitochondrial respiration or frozen in liquid nitrogen and stored at – 80 °C for further measurements of ATP content. On sampling days whole flies were also collected, put on ice, rinsed two times in ice-cold phosphate-buffered saline (PBS), and frozen in liquid nitrogen for measurements of glucose and glycogen contents as well as for pyruvate kinase (PK) and citrate synthase (CS) enzymatic activities.”

now reads,

“Following 0, 1, 2, 4, and 10 days of exposure to the HFD or to the SD, the heads and abdomens were dissected from the flies, and the resulting thorax were either directly processed for mitochondrial respiration or frozen in liquid nitrogen and stored at −80 °C for further measurements of ATP content, glycogen and glucose content and enzymatic activities.”

And the text,

“Seven flies were homogenized”.

now reads,

“Seven thorax were homogenized”.

In the original version of this Article, Figure 7 incorrectly refers to whole fly homogenates, rather than thorax muscle homogenates. As a result, the Figure legend,

“Effects of the high-fat diet (HFD) on (A) Glucose and Glycogen contents of flies, (B) Pyruvate kinase activity, and (C) Citrate synthase activity. Results are means ± s.e.m for each day of the exposure (N = 4–5 for glucose and glycogen contents; N = 6 for pyruvate kinase and citrate synthase activities) and have been analyzed using a one-way ANOVA followed by pairwise comparisons of the least-squares means using adjusted P-values (Tukey method). Dissimilar lowercase letters denote changes between days of exposure to a specifc diet, with each diferent letter(s) representing statistically significant differences (*P *< 0.05).”

now reads,

“Effects of the high-fat diet (HFD) on (A) Glucose and Glycogen contents of thorax muscle, (B) Pyruvate kinase activity, and (C) Citrate synthase activity. Results are means ± s.e.m for each day of the exposure (N = 4–5 for glucose and glycogen contents; N = 6 for pyruvate kinase and citrate synthase activities) and have been analyzed using a one-way ANOVA followed by pairwise comparisons of the least-squares means using adjusted P-values (Tukey method). Dissimilar lowercase letters denote changes between days of exposure to a specific diet, with each different letter(s) representing statistically significant differences (*P* < 0.05).”

The original Fig. 7 and its legend appear below.

**Figure 7:**
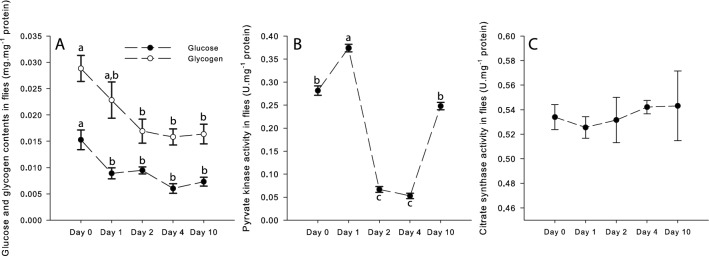
Effects of the high-fat diet (HFD) on (**A**) Glucose and Glycogen contents of flies, (**B**) Pyruvate kinase activity, and (**C**) Citrate synthase activity. Results are means ± s.e.m for each day of the exposure (N = 4–5 for glucose and glycogen contents; N = 6 for pyruvate kinase and citrate synthase activities) and have been analyzed using a one-way ANOVA followed by pairwise comparisons of the least-squares means using adjusted *P*-values (Tukey method). Dissimilar lowercase letters denote changes between days of exposure to a specifc diet, with each diferent letter(s) representing statistically significant differences (*P* < 0.05).

These changes do not affect the interpretation of the results, or the other sections of the Article, and have been implemented in the HTML and PDF versions of the manuscript.

