# Supplementary material for: Dynamic mitochondrial responses to a high-fat diet in Drosophila melanogaster
Source: Sci Rep. 2019 Mar 14;9:4531. doi: 10.1038/s41598-018-36060-5 (PMC6418259; doi:10.1038/s41598-018-36060-5)
Supplement: Supplementary file 1 — Supplementary information [file 41598_2018_36060_MOESM1_ESM.pdf]

Dynamic mitochondrial responses to a high-fat diet in *Drosophila melanogaster*.

Robert P.J. Cormier, Camille M. Champigny, Chloé J. Simard, Patrick-Denis St-Coeur, and Nicolas Pichaud.

Supplementary information.

Supplementary methods

Standard diet and high-fat diet

The standard diet contained 5 g agar-agar, 6 g sugar, 27.5 g dried yeast and 53 g cornmeal flour dissolved in 1 L of tap water, with 4 mL propionic acid, 16 mL methyl P-hydroxybenzoate [10 % w/v] added to the mixture to avoid mite and mold contamination. The high-fat diet consisted of the standard diet supplemented with 20 % (w/v) coconut oil. To determine the composition of fatty acids in the coconut oil, lipids were saponified with 400 µL of 0.5 M KOH in methanol at 100º C for 15 min, and resulting fatty acid methyl esters (FAMEs) were then prepared by adding 1 mL of 14 % BF_3 in methanol and heating at 100 ºC for 10 min. FAMEs were extracted in hexane and quantified by gas chromatography with flame ionization detection (GC/FID) using a 30 m trace-FAME column on a thermos Trace gas chromatograph (Thermo Electron Corporation, Mississauga, ON, Canada).

Permeabilization of thorax muscles

Six flies were collected and placed in a 25 mm petri dish containing 2 mL of ice-cold BIOPS solution (2.77 mM CaK_2EGTA, 7.23 mM K_2EGTA, 5.77 mM Na_2ATP, 6.56 mM $MgCl_2$, 20 mM Taurine, 15 mM Na_2Phosphocreatine, 20 mM Imidazole, 0.5 mM Dithiothreitol, 50 mM K-MES, pH 7.1). The thoraxes containing the flight muscles were dissected by removing the heads and abdomens. With sharp forceps, the thoraxes were mechanically permeabilized by inserting the tip of the forceps into the thorax and repeatedly tearing apart the tissue to obtain a loosely connected

tissue. They were then placed in 1 ml of BIOPS complemented with 12.5 μL of $5mg.mL^{-1}$ saponin solution, achieving a final concentration of 62.5 $\mu g.mL^{-1}$ of saponin, and were incubated with mild agitation on an orbital plate shaker for 20 min. The fibers were then incubated in respiration medium (120 mM KCl, 5 mM KH_2PO_4, 3 mM Hepes, 1 mM EGTA, 1 mM $MgCl_2$ (hexahydrate) and 0.2% bovine serum albumin (BSA) (w/v), pH 7.2) for 5 min. The thoraxes were then weighed and transferred into each chamber of an Oxygraph-O2K (Oroboros Instruments, Innsbruck, Austria) filled with air-saturated respiration medium with pyruvate (10 mM) and malate (2 mM).

Table S1. Fatty acid composition of the coconut oil used for the high-fat diet measured on three independent samples.

	**g / 100 g**
**Saturated Fatty Acids**	89.09 ± 0.36
**Monounsaturated Fatty acids**	8.14 ± 0.26
**Polyunsaturated Fatty Acids**	2.77 ± 0.11
**6:0**	0.65 ± 0.03
**8:0**	7.78 ± 0.25
**10:0**	5.88 ± 0.09
**12:0**	46.32 ± 0.50
**14:0**	17.36 ± 0.19
**16:0**	8.59 ± 0.18
**16:1n7**	0.02 ± 0.005
**18:0**	2.38 ± 0.08
**18:1n9**	7.71 ± 0.23
**18:1n7**	0.33 ± 0.03
**18:2n6**	2.5 ± 0.10
**18:3n3**	0.27 ± 0.01
**20:0**	0.1 ± 0.01
**20:1n9**	0.07 ± 0.003
**22:0**	0.04 ± 0.01

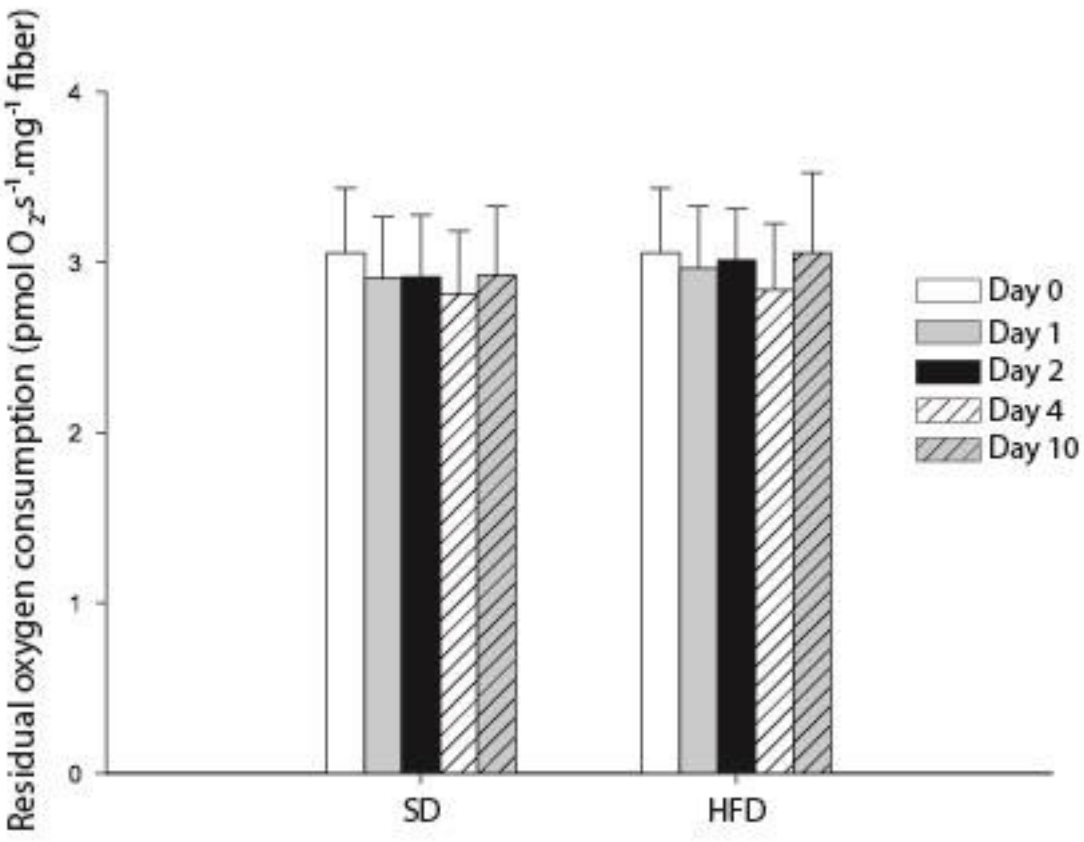

Figure S1. Residual oxygen consumption measured in permeabilized thorax of *Drosophila melanogaster* males exposed to either a standard diet (SD) or a high-fat diet (HFD). Residual oxygen consumption was measured after inhibition of Complexes I, II and III of the electron transport system by rotenone (0.5 µM), malonate (5 mM) and antimycin A (2.5 µM), respectively. Results are means ± s.e.m for each day of the exposure (N = 6).
